# Editorial: From learned helplessness to learned controllability: cognitive and neurobiological aspects involved in the development of trauma or resilience

**DOI:** 10.3389/fpsyt.2025.1692598

**Published:** 2025-09-12

**Authors:** Gustavo E. Tafet

**Affiliations:** Texas A&M University, College Station, TX, United States

**Keywords:** stress, depression, trauma, resilience, controllability, helplessness

The concept of “learned helplessness” plays a critical role in clinical psychiatry and behavioral sciences. It was originally introduced to describe a phenomenon in which individuals develop the belief that they have no control over environmental stressors (1). This concept emerged from a series of experiments in which animals were exposed to repeated inescapable and uncontrollable situations, such as receiving electric shocks without the possibility of avoidance. Later, the same animals were placed in a situation where they could avoid the shocks but, instead of attempting to escape, they simply gave up, exhibiting a passive response that reflected a sense of helplessness. When animals repeatedly experienced uncontrollable situations, they learned that their actions could not influence the outcomes, leading to a state of “learned helplessness.” Initially observed in animals, this condition was subsequently described in humans, arising from the prolonged and sustained impact of environmental stressors. When these stressors are perceived as unavoidable and uncontrollable, individuals learn to believe they lack the necessary resources to cope, resulting in learned helplessness. Consequently, this concept has been closely associated with the onset and development of psychiatric conditions, such as depression (2).

In contrast, the concept of “learned controllability” was introduced more recently to describe a condition in which individuals learn to recognize—or believe—they have the ability to respond effectively to environmental stressors, thereby counteracting the deleterious effects of learned helplessness (3, 4). Importantly, the subjective perception of controllability can be developed and learned, and it has been closely associated with the development of resilience.

This Research Topic provides novel insights into the neurobiological and psychological mechanisms underlying these concepts. By examining how the perception of control—or its absence—can influence pathophysiological processes in the context of both trauma and resilience, it offers perspectives that may help improve treatments, develop preventive strategies for trauma and depression, and foster resilience.

Understanding the mechanisms underlying these processes from cognitive and neurobiological perspectives provided essential resources for the advancement of psychiatric treatments. Modern neuroscience has revealed how the brain responds to experiences of helplessness and controllability, demonstrating cognitive and emotional processing as well as neurobiological pathways involved in neuroplasticity and psychoplasticity, which can be modulated to promote resilience or alleviate the effects of chronic stress. In this context, the articles in this Research Topic provide valuable contributions that enrich our theoretical understanding and pave the way for more precise and personalized therapeutic interventions.

A deeper understanding of how individuals perceive control could lead to the development of more effective strategies targeting the modulation of cognitive functions and neurobiological circuits involved in learned helplessness and learned controllability. By intervening in these mechanisms, it may be possible to regulate the effects of chronic stress while enhancing resilience, providing a strong foundation for more precise and personalized therapeutic approaches. In this regard, therapeutic approaches aimed at improving the subjective perception of control may help patients restructure their emotional and cognitive responses, facilitate recovery, and improve stress-coping abilities. Moreover, this Research Topic emphasizes mechanisms that facilitate positive adaptation to stress, resilience, and recovery. This perspective has the potential to contribute to clinical practice by guiding the development of interventions aimed at promoting learned controllability.

These articles collectively illuminate the multifaceted nature of stress and resilience, with particular attention to the role of control—both perceived and learned—across various contexts. Several common themes emerge, including the critical role of neural structures such as the prefrontal cortex and amygdala, and diverse neurotransmitter pathways, such as the serotonergic system, in mediating responses to stress. These contributions provide insights into how resilience can be cultivated and how the effects of stress can be regulated.

Exploring learned controllability as a modifiable factor opens new avenues for interventions aimed at enhancing resilience, particularly in individuals who have experienced significant stress, empowering them with a sense of control and agency. As research in this area progresses, it is crucial to continue investigating the neurobiological, cognitive, and emotional mechanisms underlying trauma and resilience. Future studies should also explore how early-life stress and other developmental factors interact with the neurobiological pathways of resilience.

Finally, the findings in this Research Topic highlight the need for personalized approaches to treatment that consider the unique neurobiological and psychological factors influencing each individual’s response to adverse conditions. Cross-disciplinary collaboration between neuroscientists and clinicians is essential to bridge the gap between basic research and clinical practice. By integrating insights from multiple fields, more effective, evidence-based strategies can be developed to prevent and treat stress-related disorders, ultimately fostering greater controllability and resilience in individuals exposed to chronic stressful conditions.
